# Reliability and validity of a 12-item medication adherence scale for patients with chronic disease in Japan

**DOI:** 10.1186/s12913-018-3380-7

**Published:** 2018-07-31

**Authors:** Haruka Ueno, Yoshihiko Yamazaki, Yuki Yonekura, MJ Park, Hirono Ishikawa, Takahiro Kiuchi

**Affiliations:** 1 Department of Health Communication, Graduate School of Medicine, The University of Tokyo, Tokyo, Japan; 2 Faculty of Social Welfare, Nihon Fukushi University, Mihama, Japan; 3 Graduate School of Nursing Science, St. Luke’s International University, Tokyo, Japan; 4 College of Nursing, Konyang University, Daejeon, South Korea; 5 Graduate School of Public Health, Teikyo University, Tokyo, Japan

**Keywords:** Chronic disease, Medication, Adherence, Reliability, Validity, Japan

## Abstract

**Background:**

To improve and support medication adherence among patients with chronic diseases, especially for long-term medication, it is important to consider both their relationship with healthcare providers and their lifestyle. We tested the reliability and validity of a modified 12-item Medication Adherence Scale.

**Methods:**

We revised a 14-item measure of medication adherence, created in 2009, to a more concise and clear 12-item version, and we verified the reliability and validity of the 12-item scale. We included 328 patients with chronic diseases participating in the Chronic Disease Self-Management Program in Japan from 2011 to 2014. Confirmatory factor analysis was used to assess whether the four factors assessed were the same as the previous 14-item Medication Adherence Scale. Cronbach’s coefficient alpha was used to assess internal consistency reliability, and the relationships between patient demographic characteristics and medication adherence were compared with previous studies.

**Results:**

The 12 items were categorized into the four factors “medication compliance”, “collaboration with healthcare providers”, “willingness to access and use information about medication”, and “acceptance to take medication and how taking medication fits patient’s lifestyle”. Confirmatory factor analysis showed χ^2^/df = 2.6, CFI = 0.94, and RMSEA = 0.069. Cronbach’s alpha for the 12-item scale was 0.78. Cronbach’s alpha for the four subscales was 0.74, 0.81, 0.67, and 0.45. Higher medication adherence was significantly associated with being a female patient, living with someone else, and age 40–49 years versus age 20–29 years. These relationships were the same as in previous studies.

**Conclusions:**

We modified our original 14-item scale to a 12-item Medication Adherence Scale for patients with chronic diseases, which considers their relationship with healthcare providers and lifestyle. Refinement might be needed because of the relatively low reliability of subscales. However, the modified scale is expected to contribute to more effective self-management of medication and to improving medication adherence, particularly among patients with chronic diseases who require long-term medication not only in Japan but also in other countries.

**Electronic supplementary material:**

The online version of this article (10.1186/s12913-018-3380-7) contains supplementary material, which is available to authorized users.

## Background

The number of patients with chronic diseases, such as diabetes and cardiovascular disease, is increasing in developed countries [1]. Increasing severity of chronic diseases causes greater medical expenses [2] and poorer quality of life [1]. Patients with long-term conditions also need support to prevent problems from developing and avoid having to manage complications [3].

Medication is very important for the treatment of chronic diseases. However, many patients have challenges with maintaining constant medication regimens according to instructions [4, 5], which results in low medication compliance rates [6–8]. The average level of medication adherence among patients with chronic diseases in developed countries is only 50% [7, 9, 10].

A report on patients with chronic diseases, such as diabetes and hypertension, showed that medication was only effective in one-third of patients because it was incorrectly administered [11, 12]. Other reasons why medication may be ineffective include unnecessary preventive medication [5, 13] and incorrect prescribing. For example, patients are given short-term treatment when in fact long-term medication is needed [5].

According to Morisky, correct ongoing use of medication requires patients to understand the necessity of their medication [6, 14] as well as the risks of their disease and why medication is important [5, 14]. According to Hulka and Svensson, good patient–healthcare provider relationships [15, 16] are important for medication adherence. Haynes stated improving medication adherence requires adequate social support [17]. The World Health Organization (WHO) highlights the need for patient consent to and participation in their treatment [5]; this is similar to Kamishima, who pointed out the importance of patient agreement with their treatment [18]. These reports suggest that it is crucial to clarify the various psychosocial factors related to self-management of medication in patients with chronic diseases. This is considered to be beneficial for supporting the ongoing use of medication by people living with chronic diseases [5, 6].

The WHO has suggested that discussion between patients and healthcare providers is an important psychosocial aspect of medication support and treatment decision-making [1]. The WHO guidelines note that promoting patient participation depends on valid and reliable measurement of the adherence construct. Patients’ participation in decisions about medication requires good patient–healthcare provider communication. Strong emphasis is placed on the need to differentiate adherence from compliance. The main difference is that adherence requires the patient’s agreement with recommendations. The adherence concept has adopted the definition of adherence to long-term therapy as “the extent to which a person’s behavior corresponds with agreed recommendations from a healthcare provider” [5].

In Japan, Kamishima defined medication adherence as “the extent to which patients understand their diseases and treatment thoroughly, participate positively, and accomplish their medication behavior in line with agreed recommendations”. This definition attempts to combine behavioral and psychological aspects [18]. Kamishima also stated that ideal medication use, based on her concept of adherence, is when patients continue to take their medication because they understand that it is necessary, in consultation and collaboration with their healthcare provider about managing their physical condition [18].

Medication self-management in daily life requires not only patient’s understanding of the need for medication but also good partnerships between the patient and health care provider, choice of the medication that fits into the patient’s lifestyle, and their willingness to take the medication. The presence of all these factors is part of the concept of adherence [19].

A recent systematic review of adherence scales covered 43 validated scales [20]. Some tools shed light on barriers to adherence such as patient–healthcare provider relationships, self-efficacy, patient’s lifestyle and commitment. The focus of these past scales was not only on compliance but also knowledge, and psychological factors that affected expectations and rejection of medication [6, 14, 21]. However, there are no validated tools to measure the comprehensive concept of medication adherence, including psychosocial factors related to medication behavior, and particularly patient–caregiver relationships and lifestyle factors together.

Therefore, existing scales are insufficient to capture psychosocial aspects including daily life situations and lifestyles, as well as the relationship with the medical provider, to foster effective and continuing support. Considering self-management of medication adherence in daily life by focusing on social aspects, like daily life situation and lifestyle in addition to the psychological side, measurement of the patient’s medication situation can be made from a more multifaceted viewpoint. We therefore developed a 14-item Medication Adherence Scale [19] (see Additional files 1 and 2), to include not only compliance but also psychosocial factors related to medication behavior, and particularly patient–caregiver collaboration and relationship as well as patient lifestyle. Data collection and a survey were performed in 2009, and the results were published in the Journal of Japan in 2014. This 14-item scale was recognized by various hospital officials and researchers as a measure of medication adherence in Japan. Positive feedback of this measure has been received on an informal basis. Based on the results of analysis when considering the reliability and validity of the 14-item scale, it was thought that some items required modification, to improve the clarity and convenience of the scale. From the results of testing, some items implied a double negative and were difficult for target patients to understand. The inclusion of such items lowered the subscale alpha coefficient. We realized there was a need to further clarify the wording. Because double loading was found in factor analysis, items that were similar to other items were deleted. In addition, some question assessed concepts of another subscale. Therefore, we revised the scale to improve ease of use and accuracy of measurement; we modified the 14-item scale to create the 12-item Medication Adherence Scale. This scale included “medication compliance” and added the two psychosocial factors related to medication behavior: patient–caregiver collaboration, and daily lifestyle. The 12 items were expected to fit into the original four categories, “medication compliance”, “collaboration with healthcare providers”, “willingness to access and use information about medication”, and “acceptance to take medication and how taking medication fits patient’s lifestyle”.

In this study, we modified our original 14-item scale to a 12-item Medication Adherence Scale, with some items more clearly worded, to more accurately measure medication adherence in patients with chronic diseases, and we examined the reliability and validity of the 12-item scale.

## Methods

We followed a three-step process in this study.

### Step 1. Development of the 14-item medication adherence scale and modification to the 12-item scale

Scale items were constructed based on a literature review and interviews with patients who had chronic diseases as well as prescribing physicians. We grouped the main chronic diseases into six groups: type 1 diabetes, type 2 diabetes, rheumatic diseases (including rheumatic disease and connective tissue disease), hypertension, dyslipidemia, and other (heart disease; and allergies including asthma, atopic dermatitis, and allergic rhinitis). A self-administered questionnaire including these scale items was administered to 888 patients recruited from hospital outpatients and groups of patients with chronic diseases. We analyzed 509 responses (response rate, 57.3%). In 2009, we developed a 14-item Medication Adherence Scale [19] (see Additional files 1 and 2). From the results of evaluation of the 14-item scale to verify its reliability and validity, we found it was necessary to modify some items to more accurately and conveniently measure adherence.

In 2010, we held several conferences joined by seven researchers, two patients with chronic diseases, and one employee at a pharmaceutical company to discuss the results of analysis of the 14-item Medication Adherence Scale [19]. We examined the scale’s content validity and how we could make the scale more accurate and convenient to use. We included the same four assessment factors as in the original 14-item version in a new, 12-item version of the Medication Adherence Scale: “collaboration with healthcare providers”, “willingness to access and use information about medication”, “acceptance to take medication and how taking medication fits patient’s lifestyle”, and “medication compliance”. We felt that the four core factors should not be changed.

From the 14 items, we reduced the five items on the subscale “willingness to access and use information about medication” to three items. The reason for deleting item 4) is that it was similar to item 1). In factor analysis, we found double loading of factor 1) of 0.427 factor loading and factor 2) of 0.513 factor loading. The reason for deleting item 7) is because the question assessed the patient’s ability to take measures rather than their ability to collect and use information.

We then modified any items on the 14-item scale that had double negatives. Item 14) was a double negative, and we felt that some target patients may not have properly understood the meaning of the question; it was modified to item 3) on the 12-item scale.

Next, we modified the scale to ensure that suitable answers were provided for all respondents. It may have been difficult for target patients who live alone or had few people around them, or for those who do not need support, to respond to item 11) on the 14-item scale. This was also subject to environmental factors. Inclusion of this item lowered the subscale α coefficient. Therefore, we revised the question to be appropriate for all respondents.

Finally, we modified the wording of the response options to make it easier for respondents to answer questions.

### Step 2. Reliability and validity of the 12-item medication adherence scale

During the study period, from June 2011 to June 2014, a self-administered questionnaire was sent to a total 540 patients with chronic diseases, who were participating in the Chronic Disease Self-Management Program(CDSMP) in Japan. CDSMP is the program developed at Stanford University and participants took a Japanese version of the program. We included any patients aged over 20 years old, who had a chronic disease and was continuously taking long-term medication. We excluded any patients with over 10% missing information, and those who were not taking any medication or were hospitalized at the time of the study. The questionnaire included the 12-item Medication Adherence Scale, categorized according to the four assessment factors, each of which contained three items. Each item was rated on a five-point Likert-type scale, with answers ranging from 1 (never) to 5 (always). Scores for the items on each subscale were summed to give a subscale score and an overall medication adherence score was also calculated by adding all 12 items. Scores were reversed for items 3) and 12), such that higher scores indicated higher medication adherence.

### Step 3. Statistical analysis

The mean and standard deviation were calculated for each item on the 12-item Medication Adherence Scale. Ceiling and floor effects were also investigated for each item on the 12-item scale. Confirmatory factor analysis was used to assess the scale and confirm that the theoretical four-factor model would achieve the best fit for patients with chronic diseases in Japan. Model fitness was assessed using the maximum likelihood method, with the comparative fit index (CFI) and root mean square error of approximation (RMSEA). The model was built using items from the four subscales as observed variables. Cronbach’s coefficient alpha was calculated to assess the internal consistency reliability of each subscale. The construct validity was examined for relationships by comparing patient demographic characteristics and medication adherence with previous studies. Differences between two groups according to sex, education level, marital status, and number of diagnoses were evaluated using a *t*-test. Ages were categorized into six 10-year intervals using one-way analysis of variance and Tukey’s multiple comparison test.

All analyses were conducted with SPSS version 21.0 (IBM SPSS Japan Inc., Tokyo, Japan), except for the confirmatory factor analysis, which was done using AMOS version 21.0 (IBM SPSS Japan Inc., Tokyo, Japan).

## Results

A self-administered questionnaire was sent to a total of 540 patients with chronic diseases in the community, all of whom were participating in the Chronic Disease Self-Management Program in Japan. In total, 392 patients returned the questionnaire, and 328 of these were suitable for analysis (response rate of 60.7%).

### Characteristics of respondents to the 12-item medication adherence scale

The sociodemographic and clinical characteristics of study participants are shown in Table 1. There were 72 male (22.0%) and 254 female participants (77.4%); two participants (0.6%) did not specify their sex. The mean age was 48.72 ± 13.8 years. Mean disease duration was 14.18 ± 13.0 years. Table 2 shows the means and standard deviations for each item of the scale. A ceiling effect was found for five of the 12 items on the scale: items 1), 2), 3), 6) and 11).

**Table 1:** Demographic and clinical characteristics of participants (*n* = 328)

			Number	Percent
Sex	Male		72	22.0
	Female		254	77.4
	Not specified		2	0.6
Age (years)	Mean (range)		48.72	(21–80)
Schooling	High school or less		111	33.8
	College or more		209	63.7
	Unknown		8	2.5
Marital status	Living with someone else		165	50.3
	Living alone		161	49.1
	Unknown		2	0.6
Number of diagnoses	One		166	50.6
	More than two		154	47.0
	Unknown		8	2.4
Disease duration (years)	Mean (± SD)		14.18 ±	13.0
Diagnoses of participants	Total diagnoses^a^	(*N* = 328)	As single diagnosis	(*N* = 166)
Diagnoses	Number	%	Number	%
Diabetes	48	15.2	19	11.4
Type 1	20	6.1	13	7.8
Type 2	14	4.3	4	2.4
Others	12	3.7	1	0.6
Unknown	2	0.6	1	0.6
Rheumatic disease Group^b^	99	30.2	40	24.1
Hypertension	55	17.2	4	2.4
Dyslipidemia	27	8.4	1	0.6
Heart disease^c^	12	3.7	5	3.0
Allergies^d^	48	15.2	4	2.4
Others	179	54.6	92	55.4

^a^Includes both single and one of several diagnoses

^b^Includes rheumatic disease and connective tissue disease

^c^Includes vascular and cardiovascular disease

^d^Includes asthma, atopic dermatitis, and allergic rhinitis

**Table 2:** Scores for items on the 12-item Medication Adherence Scale (n = 328)

	Mean	SD
Medication compliance	13.31	2.35
1) Over the past 3 weeks, I have taken the prescribed daily dosage of my medication.	4.59	0.84
2) Over the past 3 weeks, I have followed the instructions about when or how often to take my medication.	4.47	0.89
3) I have stopped taking medication based on my own judgment (not including times when I forgot to take my medication)	4.26	1.14
Collaboration with healthcare providers	11.16	2.82
4) I feel comfortable asking my healthcare provider about my medication.	3.63	1.13
5) My healthcare provider understands when I tell him/her about my preferences in medication taking.	3.62	1.10
6) My healthcare provider understands when I explain to him/her about my past medication including previous allergic reactions.	3.91	1.08
Willingness to access and use information about medication	10.97	2.50
7) I understand both the effects and the side effects of my medication.	3.86	0.91
8) I report side effects, allergic reactions, or unusual symptoms caused by the medication.	3.87	1.05
9) I personally search for and collect information that I want about my medicine.	3.23	1.23
Acceptance to take medication and how taking medication fits patient’s lifestyle	11.43	2.14
10) I accept the necessity of taking medication in the prescribed manner to treat my illness.	4.09	0.84
11) Taking medication is part of my everyday life, just like eating or brushing my teeth.	4.30	0.93
12) I sometimes get annoyed that I have to keep taking medicine every day.	3.03	1.28

### Confirmatory factor analysis

**Fig. 1:**
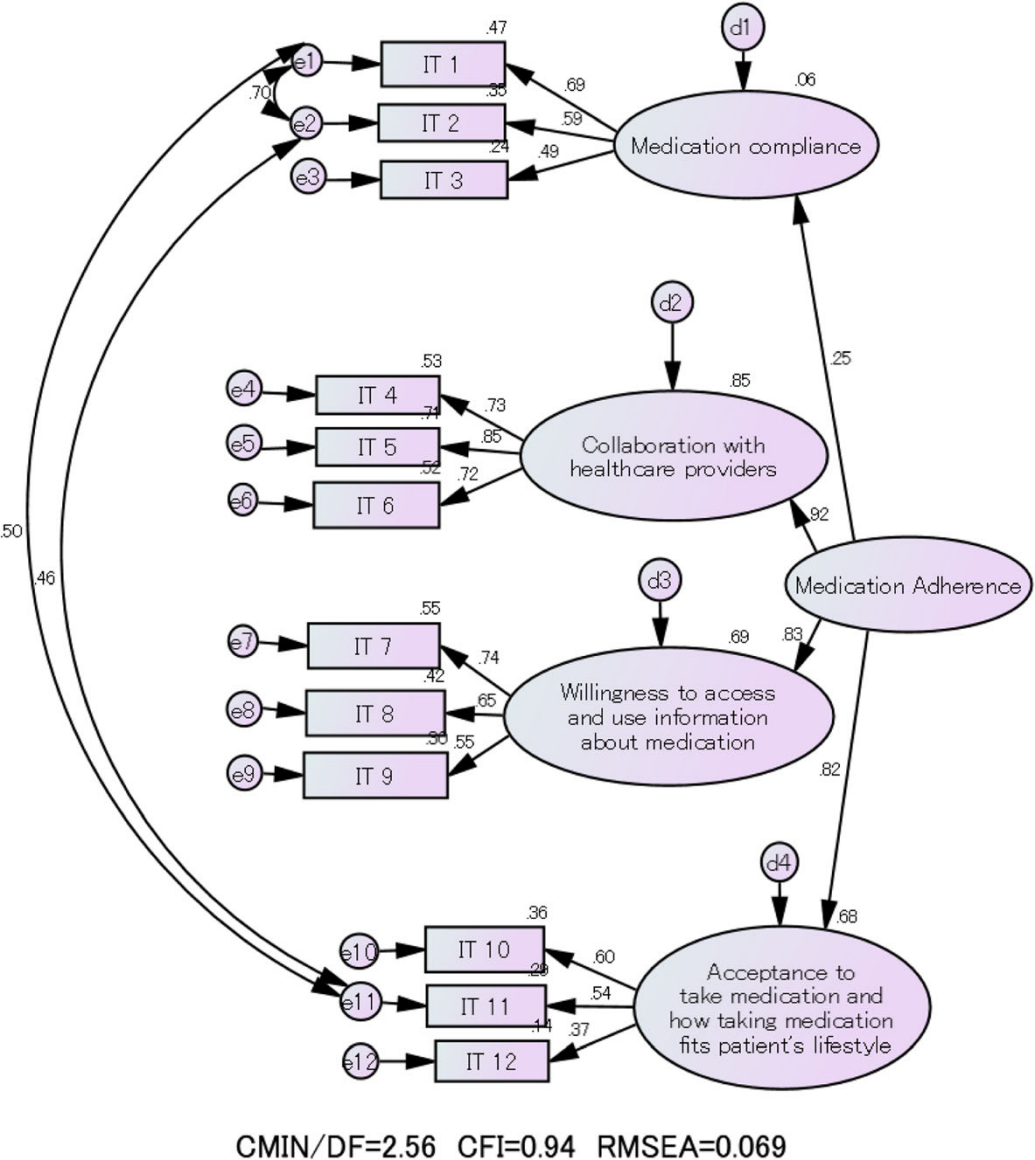
Results of the confirmatory factor analysis

The results of confirmatory factor analysis are shown in Fig. 1. The model fit indices were CFI = 0.94 and RMSEA = 0.069. There was relatively good fit between the four-factor model and the observed data. The CFI value of 0.94 was higher than 0.90, indicating a relatively good fit. The RMSEA value of 0.069 was in the reasonable fit range of 0.05–0.08.

### Internal consistency

The internal consistency of the 12-item scale was good: Cronbach’s coefficient alpha was 0.78. Cronbach’s alpha was 0.74 for the “medication compliance” subscale, 0.81 for “collaboration with healthcare providers”, 0.67 for “willingness to access and use information about medication”, and 0.45 for “acceptance to take medication and how taking medication fits patient’s lifestyle”.

### Construct validity

The relationships between patient demographic characteristics and medication adherence were comparable with previous studies, suggesting good construct validity. Table 3 shows the patient demographic characteristics associated with each subscale of the 12-item scale. Significant differences were seen between male and female, with scores for female being significantly higher on the “medication compliance” (*p* = 0.035) and “willingness to access and use information about medication” (*p* = 0.007) subscales and the total score (*p* = 0.010). For the “willingness to access and use information about medication” subscale, scores for participants aged 20–29 years were significantly lower than for those aged 40–49 years (*p* = 0.027). There were no significant correlations with single or multiple diagnoses.

**Table 3:** Correlation coefficients between total score and four subscales of the 12-item Medication Adherence Scale (*n* = 328)

	Medication compliance				Collaboration with healthcare providers				Willingness to access and use information about medication				Acceptance to take medication and how taking medication fits patient’s lifestyle				Total score			
	*n*	Mean	(SD)	*p* ^a^	*n*	Mean	(SD)	*p* ^a^	*n*	Mean	(SD)	*p* ^a^	*n*	Mean	(SD)	*p* ^a^	*n*	Mean	(SD)	*p* ^a^
Male	72	12.71	2.81	**0.035**	72	10.82	2.74	0.265	71	10.14	2.95	**0.007**	72	11.43	2.14	0.985	71	45.00	7.56	**0.010**
Female	252	13.48	2.18		244	11.24	2.84		250	11.18	2.31		254	11.43	2.15		239	47.37	6.45	
20–29	21	13.24	2.19	0.083	21	11.29	2.22	0.806	20	9.80	2.14	**0.027**	21	11.05	1.99	0.770	20	45.10	4.88	0.745
30–39	57	13.19	2.38		57	11.19	3.31		58	11.19	2.84		58	11.40	2.39		56	47.11	8.30	
40–49	77	12.79	2.73		77	11.44	2.44		77	11.62	2.22	^b^40–49>	78	11.46	1.98		76	47.50	6.31	
50–59	61	13.49	2.20		58	11.31	2.65		61	10.66	2.78	20–29	61	11.56	2.31		58	46.98	6.37	
60–69	45	14.04	1.71		43	10.65	3.12		43	10.53	1.97		45	11.53	2.02		41	46.34	6.24	
70+	26	13.73	1.91		23	11.13	3.14		25	10.64	2.75		26	12.00	2.06		22	47.82	7.42	
Living with someone else	163	13.63	2.26	**0.013**	162	11.22	2.65	0.711	162	11.01	2.34	0.840	165	11.56	2.07	0.275	158	47.41	6.44	0.151
Living alone	161	12.98	2.41		154	11.10	3.01		159	10.95	2.65		161	11.30	2.22		152	46.30	7.13	
High school or less	110	13.49	2.21	0.335	105	10.81	3.06	0.144	110	10.64	2.70	0.118	111	11.38	1.90	0.796	104	46.37	6.49	0.406
College or more	208	13.22	2.45		205	11.31	2.71		205	11.12	2.37		209	11.44	2.26		200	47.05	6.96	
Single diagnosis^c^	164	13.16	2.47	0.243	163	11.25	2.74	0.553	163	10.87	2.53	0.495	166	11.60	2.14	0.194	159	46.80	6.80	0.886
Multiple diagnoses	154	13.47	2.23		148	11.05	2.94		152	11.06	2.51		154	11.29	2.13		146	46.91	6.81	

Excluding any missing values. Significant values (*p* < 0.05) are highlighted in **bold**

^a^Differences between two groups were evaluated by *t*-test, and between three or more groups by one-way analysis of variance

^b^Differences in mean values were assessed using Tukey’s multiple comparison test

^c^A single diagnosis included multiple diagnoses within the same disease group; multiple diagnoses included any outside the disease group of rheumatic disease and allergies

## Discussion

In this study, we modified and validated a 12-item Medication Adherence Scale, to improve and support medication adherence among patients with chronic diseases, especially for long-term medication. It is important to consider both patients’ relationship with healthcare providers and their lifestyle. This 12-item Medication Adherence Scale is a modified version of our original 14-item scale, with four subscales or factors, including “medication compliance” and three new factors: “collaboration with healthcare providers”, “willingness to access and use information about medication”, and “acceptance to take medication and how taking medication fits patient’s lifestyle”. These factors are significant because they represent the range of difficulties in ensuring long-term patient medication adherence.

In assessing the reliability and internal consistency of the 12-item Medication Adherence Scale, the results for the subscales were adequate. Values ranged from 0.67 to 0.81, except for “acceptance to take medication and how taking medication fits patient’s lifestyle”, where Cronbach’s coefficient alpha was 0.45. By excluding item 12), “I sometimes get annoyed that I have to keep taking medicine every day”, the Cronbach’s alpha for this subscale was 0.53.

We excluded item 12) from the 12-item scale so that all participants would be able to respond; the previous item was not applicable to some respondents. It is possible for the Cronbach’s coefficient alpha to change for different participant populations, so we did not exclude item 12) in the new version. We do, however, suggest that it may be inadvisable to use this subscale on its own, even though it provides a helpful contribution to the overall Medication Adherence Scale.

The results of the confirmatory factor analysis indicated that the 12-item Medication Adherence Scale fit the four-factor model, modified from the model that considered three pairs of error correlations. The path coefficient for the subscale on “medication compliance” (0.25) was lower than the other three subscales. We suggest that this subscale is unique of the concept of overall medication adherence compared to the other three subscales. The path coefficient of the subscale “acceptance to take medication and how taking medication fits patient’s lifestyle” was 0.82, a high value despite the low Cronbach’s alpha, which indicated lower internal consistency. This suggests that this subscale is an essential component in the concept of medication adherence.

The relationships between patient demographic characteristics and medication adherence were comparable with previous studies, suggesting good construct validity. Significant differences were seen between male and female for the “medication compliance” and “willingness to access and use information about medication” subscales and total scores, with female scoring significantly higher than male. Participants aged 20–29 years had significantly lower scores than those aged 40–49 years. Previous studies also showed that female and older age showed higher medication adherence [22]. Younger patients with less severe conditions often found it more difficult to continue medication [23]. There were significant lifestyle differences between participants aged 20–29 and 40–49 years, which may affect willingness to access and use information about medication. Older patients may also have had their diseases longer and may therefore be more used to their medication [24, 25]. We also found that people who lived with someone else were more likely to have higher medication adherence, which is consistent with previous studies reporting that living with someone else or having family support were linked to higher medication adherence [5, 17].

The use of this scale is considered appropriate not only for Japan but also for international use. Patients may use this 12-item Medication Adherence Scale to self-check the fit between lifestyle and medication. For healthcare providers, the 12-item scale offers three advantages. The first advantage is it may be used to capture the items or subscales that may be difficult for patients when their living circumstances change, for example, if they are hospitalized or enter into a nursing home. This may help us better understanding patients’ medication adherence, and eventually will enable us to provide better support for them. Second, this scale allows healthcare providers to identify those factors that are particularly difficult for patients. This could help patients and healthcare providers more collaboratively work and share information about medication. Third, this scale enables comparisons of medication adherence at different points of treatments over time and it also makes assessment of intervention effects easier. Together, this new 12-item scale is expected to help those in Japan and overseas with chronic diseases more effectively self-management their medication as well as improve the quality of their life and long-term health outcomes. In the future, this scale is expected to be useful not only for research but also in practice as a convenient evaluation index for medication adherence in patients with a variety of chronic diseases.

There are some limitations to this study. First, the participants in this study were patients with chronic diseases participating in the Chronic Disease Self-Management Program in Japan. They may therefore have been more interested in their self-management than other patients in general with chronic diseases, although they may also have needed more treatment because their diseases were in more serious condition. There may have been selection bias in the recruitment stage. Participants with type 2 diabetes, hypertension, and dyslipidemia were not an average sample of patients with these diseases; our participants were more aware of and more likely to adhere to their medication regimes. The study participants had a range of disease severity. Some had multiple diagnoses, and their disease severity threatened their activities of daily life. Other participants had less severe disease that did not affect them in daily life. It was therefore impossible to compare between diseases in this study.

Second, this scale was implemented in a limited population of Japan. There is a need to consider replicating the study for international or future use. Third, we assessed the scale, including the ceiling effect, but focused on content validity. Further research is needed to select participants with more illnesses in common and with less variation, to improve comparisons. Fourth, the scale was not compared with other quantitative measures that have been validated elsewhere. Finally, this study was limited to examining validity and reliability. Further examination of test-retest reliability and external validity are needed.

## Conclusions

This study has demonstrated the reliability and validity of the 12-item Medication Adherence Scale for patients with chronic disease in Japan. The scale was categorized into four factors: “medication compliance”, “collaboration with healthcare providers”, “willingness to access and use information about medication”, and “acceptance to take medication and how taking medication fits patient’s lifestyle”. These reflect not only compliance but also patients’ relationships with healthcare providers and their lifestyles. This scale may be used to support more effective medication self-management, as it provides a convenient way to assess the medication-taking behavior of patients with chronic diseases. The scale is therefore expected to contribute to improving patients’ quality of life and health care outcomes through better adherence to medication regimes.

## Additional files

Additional file 1: Medication Adherence Scale 14-item Version(Original Version). Abstract of 14-item Version. (PDF 109 kb)

Additional file 2: Medication Adherence Scale 14-item Version(Original Version). Original 14-item Scale. (PDF 93 kb)

## Abbreviations

CFI: Comparative fit index
RMSEA: Root mean square error of approximation
χ^2^/df: Chi-squared / degrees of freedom

## References

[1] World Health Organization. Global status report on noncommunicable diseases 2014. http://apps.who.int/iris/bitstream/10665/148114/1/9789241564854_eng.pdf?ua=1. Accessed 8 Jun 2018. 10.1161/STROKEAHA.115.008097 25873596

[2] Cleemput I, Kesteloot K, DeGeest S (2002). A review of the literature on the economics of noncompliance. Room for methodological improvement. Health Policy.

[3] World Health Organization. Preventing chronic diseases: a vital investment. http://www.who.int/chp/chronic_disease_report/contents/en/. Accessed 8 Jun 2018.

[4] Horne R, Weinman J (1999). Patients’ beliefs about prescribed medicines and their role in adherence to treatment in chronic physical illness. J Psychosom Res.

[5] World Health Organization. Adherence to long-term therapies: Evidence for action. http://apps.who.int/medicinedocs/en/d/Js4883e/1.html. Accessed 8 Jun 2018.

[6] Morisky DE, Green LW, Levine DM (1986). Concurrent and predictive validity of a self-reported measure of medication adherence. Med Care.

[7] Sackett DL, Haynes RB, Gibson ES, Taylor DW, Roberts RS, Johnson AL (1978). Patient compliance with antihypertensive regimens. Patient Couns Health Educ.

[8] Haynes RB, Haynes RB, Taylor DW, Sackett DL (1979). Determinants of compliance: the disease and the mechanics of treatment. Compliance in health care.

[9] Green CA (1987). What can patient health education coordinators learn from ten years of compliance research?. Patient Educ Couns.

[10] Nieuwlaat R, Wilczynski N, Navarro T, Hobson N, Jeffery R, Keepanasseril A (2014). Interventions for enhancing medication adherence. Cochrane Database of Systematic Reviews.

[11] Liebl A, Neiss A, Spannheimer A, Reitberger U, Wagner T, Gortz A (2001). Costs of type 2 diabetes in Germany. Results of the CODE-2 study. Dtsch Med Wochenschr.

[12] Liebl A, Neiss A, Spannheimer A, Reitberger U, Wieseler B, Stammer H, Goertz A (2002). Complications, co-morbidity, and blood glucose control in type 2 diabetes mellitus patients in Germany--results from the CODE-2 study. Exp Clin Exp Clin Endocrinol Diabetes.

[13] DiMatteo MR, Haskard KB, Williams SL (2007). Health beliefs, disease severity, and patient adherence: a meta-analysis. Med Care.

[14] Morisky DE, Ang A, Krousel-Wood M, Ward HJ (2008). Predictive validity of a medication adherence measure in an outpatient setting. J Clin Hypertens.

[15] Hulka BS, Haynes RB, Taylor DW, Sackett DL (1979). Patient-clinician interactions and compliance. Compliance in health care.

[16] Svensson S, Kjellgren KI, Ahlner J, Saljo R (2000). Reasons for adherence with antihypertensive medication. Int J Cardiol.

[17] Haynes RB, McDonald HP, Garg AX (2002). Helping patients follow prescribed treatment: clinical applications. JAMA.

[18] Kamishima S, Noji A, Katakura Y, Maruyama T (2008). Factors related to adherence to a medication regimen in out-patients being treated for stroke. J Jpn Acad Nurs Sci.

[19] Ueno H, Yamazaki Y, Ishikawa H (2014). Reliability and validity of medication adherence scale for patients with chronic disease in Japan. Jpn J Health Educ Promotion.

[20] Nguyen T-M-U, Caze AL, Cottrell N (2013). What are validated self-report adherence scales really measuring?: a systematic review. Br J Clin Pharmacol.

[21] Hirastuka S, Kumano H, Katayama J, Kishikawa Y, Hishinuma T, Yamauchi Y, Mizugaki M (2000). Drug compliance scale 1. Development of the drug compliance scale. Yakugaku Zasshi.

[22] Krousel-Wood M, Thomas S, Muntner P, Morisky D (2004). Medication adherence: a key factor in achieving blood pressure control and good clinical outcomes in hypertensive patients. Curr Opin Cardiol.

[23] Grant RW, O’Leary KM, Weilburg JB, Singer DE, Meigs JB (2004). Impact of concurrent medication use on statin adherence and refill persistence. Arch Int Med.

[24] Wang Y, Lee J, Toh MPHS, Tang WE, Ko Y (2012). Validity and reliability of a self-reported measure of medication adherence in patients with type 2 diabetes mellitus in Singapore. Diabet Med.

[25] Michetti P, Weinman J, Mrowietz U, Smolen J, Peyrin-Biroulet L, Louis E (2017). Impact of treatment-related beliefs on medication adherence in immune-mediated inflammatory diseases: results of the global ALIGN study. Adv Ther.

