# Supplementary material for: Reliability and validity of a 12-item medication adherence scale for patients with chronic disease in Japan
Source: BMC Health Serv Res. 2018 Jul 31;18:592. doi: 10.1186/s12913-018-3380-7 (PMC6069892; doi:10.1186/s12913-018-3380-7)
Supplement: Supplementary file 1 — Medication Adherence Scale 14-item Version(Original Version). Abstract of 14-item Version. (PDF 109 kb) [file 12913_2018_3380_MOESM1_ESM.pdf]

Additional file 1: Medication Adherence Scale 14-item Version（Original Version）

Reliability and validity of medication adherence scale for patients with chronic disease in Japan

Haruka UENO*[1], Yoshihiko YAMAZAKI*[2], Hirono ISHIKAWA*[1]

Abstract

Objective: Medication adherence among patients with chronic diseases should be considered in the context of their relationship with healthcare providers and lifestyles. To support medication adherence among patients with chronic diseases under long-term medication, we developed a new medication adherence scale and evaluated its reliability and validity.

Methods: The scale items were constructed based on a literature review and interviews with patients with chronic diseases and prescribing physicians. A self-administered questionnaire including these scale items was administered to 888 patients recruited from hospital outpatients and groups of patients with chronic diseases. The study analyzed 509 responses (response rate＝57.3%).

Results: In exploratory factor analysis, 14 items were categorized under 4 factors (“collaboration with healthcare providers,” “motivation of access and utilization of information regarding medication,” “agreement with taking medication and its fit with their lifestyle,” and “medication compliance”). A confirmatory factor analysis showed that χ^2/df＝4.4, CFI＝0.93, and RMSEA＝0.047. The Chronbach’s α of the subscales were 0.92, 0.80, 0.55 and 0.74, respectively. The correlation coefficients between these subscales and other related measures were between 0.43 and 0.60, indicating adequate concurrent validity. The relationships between patient demographic characteristics and medication adherence were comparable with previous studies, suggesting good constructive validity.

Conclusions: This study demonstrated the reliability, validity and practicality of the newly developed scale for assessing medication adherence among patients with chronic diseases. Further refinements may be needed to improve the relatively low reliability for one of the subscales.

[JJHEP, 2014;22(1):13-29]

Key words: chronic disease, medication, adherence, reliability, validity

*[1] Department of Health Communication, Graduate School of Medicine, The University of Tokyo

*[2] Faculty of Social Welfare, Nihon Fukushi University
