# Supplementary material for: Reliability and validity of a 12-item medication adherence scale for patients with chronic disease in Japan
Source: BMC Health Serv Res. 2018 Jul 31;18:592. doi: 10.1186/s12913-018-3380-7 (PMC6069892; doi:10.1186/s12913-018-3380-7)
Supplement: Supplementary file 2 — Medication Adherence Scale 14-item Version(Original Version). Original 14-item Scale. (PDF 93 kb) [file 12913_2018_3380_MOESM2_ESM.pdf]

Additional file 2: Medication Adherence Scale 14-item Version（Original Version）

Instructions

This is a survey about your use of the medication that is currently prescribed for you. (Please answer all items 1-14) Unless otherwise specified, please base your answer on your experiences over the last six months or so. If you suffer from multiple conditions, your response should reflect your overall usage of medication.

**Note: In this survey, "medication" includes medicine administered orally, injections (insulin, for example), ointments, medicated patches, and inhalants.*

To what extent do these apply? (circle the one that most applies)

(never ~ always)

	never	rarely	sometimes	often	always
Items 1)~14)	1	2	3	4	5

Questions 1-14

I. Relationship with my healthcare provider with regard to medication

1) I can freely ask my healthcare provider about medication without constraint.
2) I can share my thoughts and goals about medication with my healthcare provider.
3) I can share my past course of treatment with medication with my healthcare provider.

II. Collecting and using medication-related information

4) I have asked about anything I do not understand about the medication I am using.
5) I report side effects, allergic reactions, or unusual symptoms caused by medication.
6) I know about the medication I am using and the need to take it.
7) I am taking measures to continue medication (taking measures on a daily basis, etc.).
8) I am searching for and using the information necessary for my medication.

III. Ideas and attitude towards medication

9) I am convinced that medication is necessary.
10) Taking medication is part of my everyday life, just like eating or brushing my teeth.
11) I am not resistant to receiving help from family and people around me, such as reminding me to take my medication.

IV. Current status of medication use (please respond regarding use during this 3-week period)

12) Over the past three weeks, I have been taking medication at the prescribed daily dosage.
13) Over the past three weeks, I have been taking medication at the specified time/s.
14) I will not stop taking medication based on my own judgment.
